# A Virtual Support Program for Asian Family Caregivers of Individuals with Alzheimer’s: Practical Challenges

**DOI:** 10.1093/geroni/igaf122.2844

**Published:** 2025-12-31

**Authors:** Seulgi Ryu, Dongmi Kim, Wonshik Chee, Eun-Ok Im

**Affiliations:** The University of Texas at Austin, Austin, Texas, United States; The University of Texas at Austin, Austin, Texas, United States; University of Texas at Austin, Austin, Texas, United States; University of Texas at Austin, Austin, Texas, United States

## Abstract

The number of web-based virtual support programs supporting caregivers of individuals living with Alzheimer’s disease has grown significantly. Several studies have examined challenges in implementing culturally tailored virtual support programs for racial/ethnic minorities in the U.S. However, there remains a critical need to understand the practical challenges in implementing such programs for Asian American women family caregivers of individuals with Alzheimer’s disease. This presentation discusses the practical challenges identified in a culturally tailored virtual support program for Asian American women family caregivers. The ongoing parent study aims to develop and preliminary evaluate a virtual support program in improving the health outcomes of Asian American women family caregivers of individuals with Alzheimer’s disease. The participant recruitment, program implementation, and data collection are in progress. Individual team members’ research diaries documented key challenges, which were analyzed using basic content analysis. Four major challenges emerged: (a) maintaining engagement was challenging due to dropouts and non-responsiveness; (b) some participants primarily focused on obtaining information only, making it challenging to establish rapport and fully achieve the program goal of providing culturally tailored coaching and support; (c) some caregivers sought more specialized services, looking for professional institutions services; and (d) participants hesitated to share struggles, often withholding emotional or personal difficulties. These findings highlight key challenges in implementing a culturally tailored virtual support program among Asian American women caregivers. Addressing these challenges will be crucial in improving future virtual support programs for this population.

